# Malaria in Haiti: A descriptive study on spatial and temporal profile from 2009 to 2018

**DOI:** 10.1590/0037-8682-0355-2021

**Published:** 2022-02-25

**Authors:** Jean Ricardo Jules, Jeronimo Alencar, Martha Cecília Suárez-Mutis, Ernst Jn Baptiste, Hermano de Albuquerque, Maria Goreti Rosa-Freitas, Christian Raccurt, Ricardo Lourenço de Oliveira, Teresa Fernandes Silva-do-Nascimento

**Affiliations:** 1 Fundação Oswaldo Cruz, Instituto Oswaldo Cruz, Laboratório de Diptera, Rio de Janeiro, RJ, Brasil.; 2 Fundação Oswaldo Cruz, Instituto Oswaldo Cruz, Laboratório de Mosquitos Transmissores de Hematozoários, Rio de Janeiro, RJ, Brasil.; 3 Fundação Oswaldo Cruz, Instituto Oswaldo Cruz, Laboratório de Doenças Parasitárias, Rio de Janeiro, RJ, Brasil.; 4 Ministère de la Santé Publique et de Population (MSPP), Laboratoire et de Recherche (DELR), Direction d'Épidémiologie, Port-au-Prince, Haiti.; 5 Geniac Ltda, São Paulo, SP, Brasil.; 6 Université Quisqueya (UniQ), Port-au-Prince, Haiti.

**Keywords:** Malaria, Haiti, Descriptive study, Epidemiology

## Abstract

**Background:**

Haiti is one of the Caribbean countries where malaria persists. More than 99% of malaria cases are caused by *Plasmodium falciparum*, the main vector being the mosquito *Anopheles albimanus*. In this paper, we describe the epidemiological profile of malaria in Haiti between 2009 and 2018.

**Methods:**

We analyzed information on cases reported by the Ministry of Health of Haiti and the World Health Organization (WHO).

**Results:**

Between 2009 and 2018, 232,479 malaria cases were reported by the Ministry of Public Health and Population (MSPP); an increase in the incidence of malaria in the country in 2010, followed by a decrease in 2011, was primarily observed. Due to recent efforts to reduce malaria by 2020, its incidence declined from 60,130 cases in 2010 to 8,978 cases in 2018. Controversially, in terms of the number of reported cases, the MSPP and WHO report conflicting data. However, the results from both datasets present the same trend in Haiti from 2009 to 2018. The results also illustrate the endemicity of the disease throughout Haiti, both in rural and urban areas, especially along the coast.

**Conclusions:**

This study emphasizes the need to promote official data collection and analyses, as well as the application of epidemiological surveillance of malaria at the municipal level, for a better understanding of the real impact of malaria on the Haitian population and to create more appropriate interventions.

## INTRODUCTION

Haiti and the Dominican Republic (DR) occupy the island of Hispaniola and are the only countries in the Caribbean where malaria persists^1^. Of all reported cases of malaria on this island in 2019, more than 90% were from Haiti^2^. Malaria remains one of the top ten causes of death in Haiti^3^. In all regions of the country^4^, more than 80% of the Haitian population is at risk of malaria^2^, mainly those living below 300-m altitudes^5^. The predominant malaria parasite is *Plasmodium falciparum* ^6^ ^,^ ^7^. *Plasmodium malariae* was registered in Haitian refugees in Jamaica^8^ and, recently, in a Les Irois infant in the Sud department of Haiti (personal communication). Furthermore, some cases of *Plasmodium vivax* were confirmed in Haiti in 1946^9^ and 1983^10^. However, no study has demonstrated the autochthonous circulation of *P. vivax* in Haiti. Therefore, the transmission of *P. vivax* is considered absent in Haiti^11^ ^,^ ^12^. *Anopheles albimanus* Wiedemann is the only species that meets all malaria vector criteria in Haiti to date^2^ ^,^ ^13^. 

In 2009, Haiti and the DR developed a strategic plan to eliminate malaria on the island of Hispaniola by 2020^14^ ^,^ ^15^. Although this goal is yet to be achieved, progress has been made by strengthening prevention and treatment activities^3^. In this context, the Haitian National Health Policy presently prohibits presumptive treatment of malaria in Haiti, thereby requiring diagnostic confirmation prior to any treatment, and chloroquine, with the incorporation of primaquine, is considered to be the first-line drug for the treatment of uncomplicated malaria^16^. Prior to 2012, microscopic examination of thick and thin blood smears from suspected patients, which required trained technicians, was the only available diagnostic method for malaria in Haiti^17^. However, after the 2010 earthquake, the use of rapid diagnostic tests (RDTs) was temporarily approved for 90 days^18^ ^,^ ^19^; then, as of 2012, the Ministry of Public Health and Population (MSPP), in partnership with the Center for Disease Control and Prevention (CDC), authorized RDT as an official diagnostic method for malaria in Haiti^14^. Despite such progress, the trend in Haiti remains unknown^20^, as official data underestimate the country's disease situation in terms of morbidity and mortality. In addition, even confirmed cases of malaria have been poorly investigated. For example, in 2017, only 0.2% of the total number of confirmed cases were investigated^2^. This study aims to conduct a spatial and temporal analysis of malaria in the Republic of Haiti from 2009 to 2018, using secondary data on malaria cases reported by the MSPP and WHO. 

## METHODS

### Study area

**FIGURE 1:**
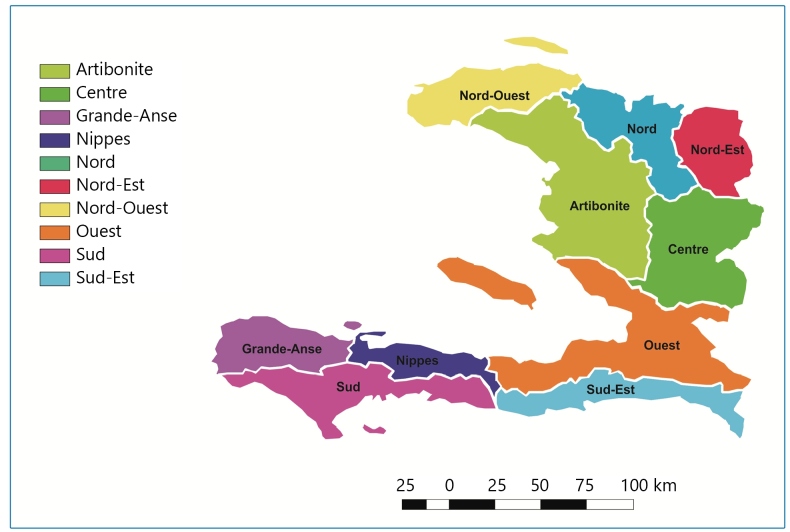
Map of Haiti displaying the ten departments.

Haiti is an Antillean country located in the western part of the island of Hispaniola. Its surface is 27,750 km², and it contains ten departments (equivalent to states or provinces): Artibonite, Center, Grand’Anse, Nippes, Nord, Nord-Est, Nord-Ouest, Ouest, Sud, and Sud-Est (Figure 1). The country’s population was estimated at 11.2 million inhabitants in 2018^21^. Due to its position in latitude, Haiti has a tropical climate, characterized by the alternation between wet (May to November) and dry seasons (November to May). Average temperatures range from 32°C in the plains and 22°C in the mountains in July (summer) to 28°C in the plains and 15°C in the mountains in January (winter)^22^. Annual precipitation varies between 400 and 3,000 mm from one region to another. Two primary rainy seasons occur from April to June and September to November. Haiti is also prone to hurricanes and seasonal tropical storms^23^. 

### Study design

The number of malaria cases reported in Haiti from 2009 to 2018 was evaluated and analyzed. This study was also conducted based on reported cases of malaria in the ten departments of Haiti. Data were obtained from MSPP statistical reports published on the website, https://mspp.gouv.ht, which can be accessed freely.

The suspected and confirmed malaria cases were registered by the public health facilities and other partners via parasitological diagnosis by thick and thin blood smears from 2009 to 2011 and via RDTs from 2012 to 2018. 

Notably, most statistical reports of the MSPP provide information on the malaria-positive lamina index and positivity rate for malaria by 10,000 inhabitants per year and per department. 

### Statistical analysis

Occurrence measures or morbidity indicators (the annual parasite index, incidence rate, and prevalence rate) and the proportion of malaria cases confirmed per department and per year were used. As previously mentioned, in some MSPP statistical reports, only the suspected cases and the proportions of the corresponding confirmed cases are displayed. As the number of cases is a discrete, quantitative variable, the following formula was applied:

$$Confirmed\;cases=\frac{Suspected\;cases}{100}x\;Proportion\;of\;confirmed\;cases$$

Results with decimal values were rounded up to the first decimal place (upward: ≥ 5; downward: ≤ 4).

### Malaria incidence rate (Ir)

 The “**Ir**” refers to the number of new malaria cases per 1,000 population of individuals at risk during a given time period. This was estimated using the following equation:

$$Ir=\frac{New\;cases\;during\;a\;given\;time\;period}{Total\;sum\;of\;people\;at\;risk\;over\;time}x1000$$

### Malaria prevalence rate (Pr)

 The “**Pr**” is the measure of the total number of existing cases (new and old) of a disease at a point or time period in a given population. This was estimated using the following equation:

$$Pr=\frac{Total\;of\;cases\;during\;a\;given\;time\;period}{Population\;during\;the\;same\;time\;period}x\;100$$

### Malaria case proportion (P%)

 The “**P%**” indicates in which areas the highest or lowest percentage of cases was registered each year. The formula is as follows:

$$P\%=\frac{Number\;of\;cases\;by\;department\;during\;the\;year}{Total\;cases\;in\;all\;departments\;during\;the\;same\;year}x100$$

### Annual parasite index (API)

The API is calculated as the number of malaria-positive patients per 1,000 inhabitants. The formula is as follows:

$$API=\frac{Total\;number\;of\;confirmed\;malaria\;cases}{Total\;resident\;population\;in\;the\;same\;year}x\;1000$$

As the WHO data only display global malaria cases, and not by Haitian departments, the calculation of API was performed only with the MSPP data. The API of each department for each year studied was integrated into a geographic information system, allowing analyses to achieve a spatial vision of malaria risk in Haiti by department and by year. For these analyses, we used ArcGIS 10 software (Environmental Systems Research Institute, Redlands, CA) and IBM SPSS Statistics 22 software (Chicago, IL, USA). 

This was a retrospective study in which descriptive analyses of the epidemiological data were performed using Excel 12.0 (Office 2007).

## RESULTS

**FIGURE 2:**
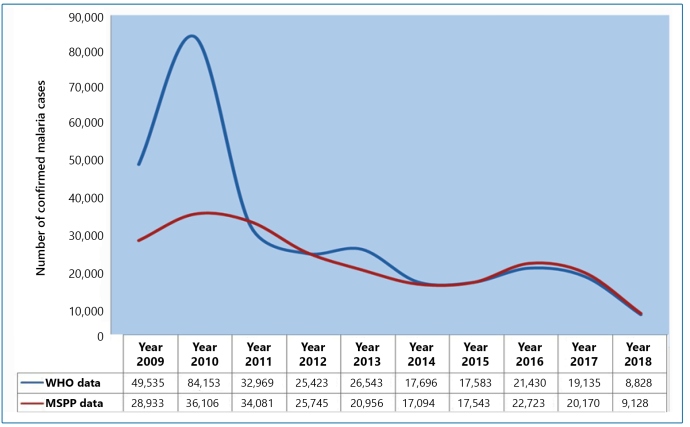
Representation of the malaria trend in Haiti, from 2009 to 2018, using MSPP and WHO data.

Between 2009 and 2018, a total of 232,479 and 303,295 confirmed malaria cases in Haiti were reported by the MSPP and WHO, respectively. Between 2010 and 2018, Haiti greatly reduced its number of malaria cases, from 84,153 cases/year to 8,828 cases/year according to the WHO data and from 36,106 cases/year to 9,128 cases/year according to the MSPP (Figure 2; Table 1). Decreases of 89.33% and 74.71% were observed by the WHO and MSPP, respectively, with an average decrease of 82.02%. Compared to 2009, the WHO data reported more than a 40% increase in 2010, followed by a small increase of 4.21% in 2013 and 17.95% in 2016. The MSPP data illustrated the same trend; however, there was a slight increase (2.55%) between 2014 and 2015 and a 22.80% increase from 2015 to 2016. 

**TABLE 1:** National malaria cases in Haiti reported by both the MSPP and WHO from 2009 to 2018.

		Tested studied individuals		Confirmed cases		Incidence rate		Prevalence		Prevalence rate	
Year	Population at risk	MSPP	WHO	MSPP	WHO	MSPP	WHO	MSPP	WHO	MSPP	WHO
2009	9,923,243	159,049	270,438	28,933	49,535	2.92	4.99	0.18	0.18	0.29	0.50
2010	10,085,214	172,937	270,427	36,106	84,153	3.58	8.34	0.21	0.31	0.36	0.83
2011	10,258,126	184,586	184,934	34,081	32,969	3.32	3.21	0.18	0.18	0.33	0.32
2012	10,413,211	164,893	167,772	25,745	25,423	2.47	2.44	0.16	0.15	0.25	0.24
2013	10,579,230	172,624	176,995	20,956	26,543	1.98	2.51	0.12	0.15	0.20	0.25
2014	10,745,665	273,707	261,403	17,094	17,696	1.59	1.65	0.06	0.07	0.16	0.16
2015	10,911,819	302,740	303,740	17,543	17,583	1.61	1.61	0.06	0.06	0.16	0.16
2016	11,078,033	339,781	302,044	22,723	21,430	2.05	1.93	0.07	0.07	0.21	0.19
2017	11,244,774	335,145	295,572	20,170	19,135	1.79	1.70	0.06	0.06	0.18	0.17
2018	11,411,527	288,294	288,249	9,128	8,828	0.80	0.77	0.03	0.03	0.08	0.08

**FIGURE 3:**
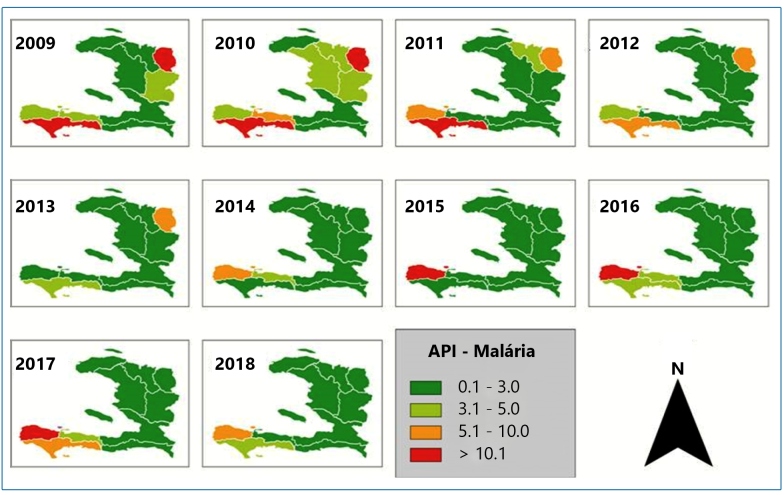
Maps of malaria transmission risk by annual parasitic index (API) 2009-2018 in the ten departments of Haiti.

The prevalence (P), prevalence rate (Pr), and incidence rate (Ir) per year, as calculated from both the MSPP and WHO data from 2009 to 2018, are presented in Table 1. The incidence rate (Ir) was equal to that of API. This is because the time period considered for the Ir calculation in this study was one year. Figure 3 shows the API per year (2009-2018) where malaria occurs in the ten departments of Haiti, with a degree of risk ranging from low (0.02 to 7.71) to medium (10.68 to 23.38).

**FIGURE 4:**
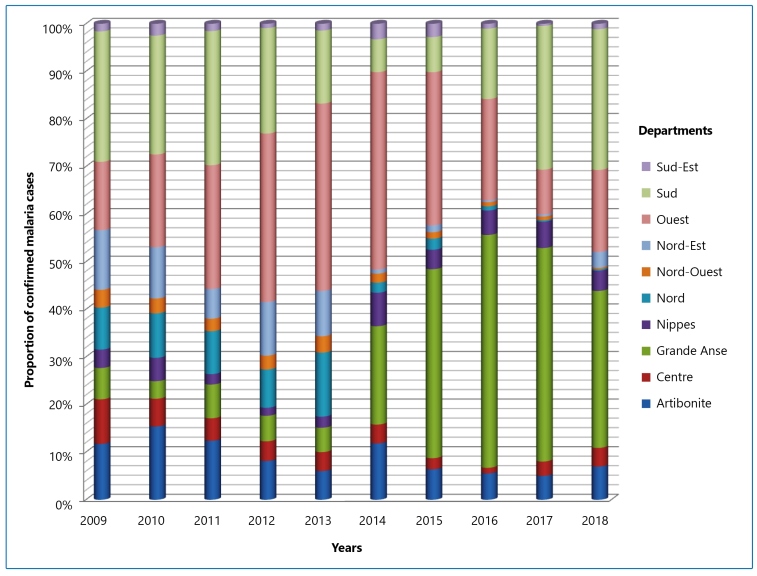
The proportion of confirmed malaria cases in Haiti by department from 2009-2018 (MSSP data).

The proportions of malaria cases are displayed in Figure 4, where we can mainly observe the concentration of malaria transmission in three of the ten departments from 2016 to 2018: Grand’Anse, Sud, and Ouest. These departments reported over 80% of all malaria cases in Haiti. The Grand'Anse, whose proportion of malaria cases did not exceed 7% per year during the period 2009-2013, experienced an elevated level of malaria transmission in 2014. Moreover, the number of malaria cases tripled in 2014 compared to 2013. In that department, a significant increase was also observed after Hurricane Matthew in 2016, with approximately half (49%) of malaria cases reported in Haiti that year. Therefore, Grand’Anse registered the highest proportion of malaria cases in 2016 during 2009-2018. Grand'Anse was the only department where there was an increase in malaria cases between 2009 and 2018 (158%). Additionally, during 2009-2017, the increase was even more pronounced (450%).

In Nord-Est, malaria cases dropped from 3,888 to 87 cases/year (a decrease of 97.76%) between 2010 and 2016 (Table 1) and from 14,952 to 942 cases/year (a decrease of 93.70%) between 2009 and 2018.

In addition to being one of the three departments of Haiti where malaria was more concentrated during the ten-year period studied, Sud registered remarkable proportions of malaria cases between 2009 and 2013 and has been explicitly ranked first during this period, as well as second place in 2012 (Figure 4). The number of malaria cases declined significantly from over 20% to less than 8% in 2014 and 2015. Although malaria cases decreased between 2014 and 2015, Sud returned to occupy third place, with more than 15% of malaria cases in 2016, and second place in 2017 and 2018, with 30% of all malaria cases registered in Haiti for the respective years.

## DISCUSSION

An overall increase in malaria incidence was reported in Haiti in 2010 compared to 2009, followed by a declining trend from 2010 to 2018 (Figure 2). Curiously, the number of malaria cases estimated by the WHO during the 2009-2018 period was higher than that reported by the MSPP. Nevertheless, both the MSPP and WHO data showed a decrease in malaria incidence from 2011 compared to 2010. Moreover, the MSPP data showed a slight increase in 2012, in contrast to the decline recorded by the WHO. Additionally, the number of malaria cases reported by the MSPP decreased in 2013; however, according to the WHO, their data revealed that the number of cases actually increased. 

A considerable difference between malaria cases reported in 2009 and 2010 by the MSPP and WHO was detected (Figure 2 **).** The data of both institutions overlap between 2011 and 2018 (post-earthquake period), except for 2013, when values deviated slightly. Therefore, the conflicting values for the first two years of the series may be due to the different Haitian sources of primary data. Even before the 2010 earthquake, Haiti faced socioeconomic problems^24^, which inevitably affected the country's health system. In 2010, in response to the earthquake, the MSPP, in collaboration with the CDC and various non-governmental organizations (NGOs), established a surveillance system for infectious diseases, including malaria^25^. The incongruence of MSPP data with those from the WHO between 2009 and 2010 may be related to deficiencies in the epidemiological surveillance system prior to the support of the CDC and NGOs. This may interfere with data collection and with updating the official MSPP database. Boncy and other collaborators^14^ noted some difficulties in the delivery of monthly laboratory reports to the central level of the MSPP, as well as the lack of communication from the central to the local structures in Haiti.

It should be noted that malaria data after the 2010 earthquake in Haiti were more robust. The reduction in conflicting data from 2011 is likely due to the MSPP’s actions in collaborating with their partners during the days and months following the earthquake. Although apparently conflicting, data from WHO and MSPP showed the same trend of malaria in Haiti during the ten-year study period. In other words, malaria declined during the 2009-2018 period, with little variation since 2011 (Figure 2).

Our analysis suggests that the Grand’Anse has always been one of the most malaria-burdened areas of Haiti; malaria cases were likely underreported in the years prior to 2014. The underreporting of malaria cases in Grand’Anse may be related to difficulties in accessing the area by land, especially in the municipalities presenting with rugged terrain^26^. Consequently, Grand'Anse, its spread, and remote municipalities remain isolated. This problem results in particularly difficult and expensive access to health care, especially in locations of high malaria burden^27^, where people must walk hours to access medical care^28^. Lack of access to health facilities can be a discouraging factor and may lead residents to use self-medication as the first alternative. It is worth noting that in Haiti, a country where the sale of medications is not regulated, anyone can buy any drug from informal vendors or pharmacies without a doctor's prescription. In any case, the surveillance and case records of malaria in this department during the analyzed period were more effective and realistic than in other departments for several reasons, such as major scale-up community case management programs, including diagnostic testing by RDTs provided by the Global Fund in 2012 to fight against acquired immunodeficiency syndrome (AIDS), tuberculosis, and malaria in Haiti^28^ ^,^ ^29^. In addition, Haiti received technical support from international malaria foundations, such as the CDC Foundation and Malaria Zero, to implement active research on malaria in the Grand’Anse in 2014. 

The significant decrease in malaria records in the Sud Department in 2014 and 2015 may be attributed to an underreporting of cases as a result of this special investment in the neighboring Grand’Anse during the same 2014-2015 period. No work has been published exploring why Sud is a malaria-prone endemic area. However, some ecological determinants may hypothetically explain the fact that Sud presents high malaria endemicity, as observed in the years prior to the passage of Hurricane Matthew in October 2016, that is, since the beginning of the series in 2009. According to MSPP^30^, Sud is considered to be one of the departments where the highest number of malaria cases has always been registered. The southern department is mainly made up of plains located along the coast, and the hydrographic network of Sud consists of 69 rivers, 250 springs, 20 ponds, one lake, and 11 lagoons^30^, which favors the formation of anopheline habitats. 

CHAI and collaborators^1^ have described the elevated risk of malaria transmission along the coast of Haiti. Malaria is mostly distributed along the coasts of the southern region of the country, which are located in both the Sud and Grand'Anse departments^31^. This coastal distribution of malaria is mainly related to the main anopheline vector bioecology, in addition to low local socioeconomic conditions. *Anopheles albimanus* is essentially a coastal mosquito and is often found in brackish water^32^. This species of anopheline is also considered a lowland mosquito^33^ that breeds in a wide variety of sunny breeding sites at altitudes below 400 m^34^.

The occurrence of Hurricane Matthew in October 2016 may be considered as the cause of the increase in malaria records in 2016 compared to 2015. Hurricane Matthew, which was a Category 5 Atlantic hurricane (maximum on the Saffir-Simpson scale) with winds reaching up to 260 km/h, caused catastrophic damage and a humanitarian crisis in Haiti; the Grand'Anse and Sud departments were the most affected ones during this period. Indeed, the after-effects of the hurricane likely included displaced human populations, environmental changes, flooding that increased the number of malaria vector larval habitats, high exposure to mosquitoes, and overcrowded human shelters. 

Most communes in the Ouest Department where malaria cases were reported are urbanized areas; two-thirds of the Haitian population live in this department. Port-au-Prince, the capital of Haiti, is located in Ouest. The department has also recorded high malaria incidence, with its municipalities accounting for 53% of malaria cases reported between 2012 and 2014^35^. However, these data seem to be misleading. Patients travel long distances to receive better health care; malaria reports are based on the health unit's location where the diagnosis occurs, not where the patient lives^36^.

In this study, by using the API to estimate malaria risk at the department level, we observed that malaria occurs in all departments of Haiti, with a degree of risk ranging from low to medium. It is known that API is influenced by population size, which grows each year regardless of whether the number of cases increases^37^. Therefore, a given area with a very low API may have a high number of cases, and vice versa. 

The use of insecticide-treated nets (ITNs) is currently the most effective means of protecting individuals against malaria. ITNs have been implemented since 2010 in almost all endemic areas of Haiti^38^. In the same context, approximately 800,000 ITNs were distributed by the Menthor Initiative, in collaboration with the United Nations Children's Fund (UNICEF), from October to December 2010^31^ ^,^ ^39^. Subsequently, approximately 2,000,000 long-lasting insecticidal nets were distributed in Haiti by the PSI in 2012. Thus, for the first time in the Americas, it was reported that a higher number of people were protected by ITNs than by indoor residual spraying, the most common method used for malaria vector control^27^. Additionally, the Global Fund to fight AIDS, tuberculosis, and malaria subsidized more than 400,000 mosquito nets in Haiti in December 2016^40^. According to the WHO, a coverage rate with impregnated mosquito nets above 80% reduces infant and juvenile mortality by approximately 25% and guarantees effective protection of more than 60% against infection^41^. On the other hand, the use of impregnated nets has been associated with major reductions in malaria morbidity and mortality in children under five in Africa^42^ ^,^ ^43^ ^,^ ^44^ ^,^ ^45^ ^,^ ^46^. Therefore, ITNs continue to be an effective tool for malaria prevention globally, especially in sub-Saharan Africa^47^. 

Considering the reduction in the number of cases between 2011 and 2018, it can be said that the mosquito net distribution campaigns contributed to the reduction of malaria cases throughout the country.

In terms of reducing malaria cases from 2009 to 2018, the most outstanding departments for malaria reduction in Haiti were Nord, Nord-Est, and Sud-Est. Nord-Est is one of the departments of Haiti where malaria was a major concern in the years prior to 2014. However, a decrease of more than 90% was observed from 2010 to 2016, and a similar observation was made in the Nord Department from 2009 to 2018. 

The considerable decrease observed in the Nord-Est department may be associated with the interventions of the binational project between Haiti and the Dominican Republic, covering the border communities of Ouanaminthe (a municipality of the Nord-Est Department) and Dajabon (a municipality in the Dominican Republic)^15^.

When comparing data between 2009 and 2018, the Nord Department showed the greatest reduction in malaria incidence among all departments of Haiti. Until now, no systematic literature review has reported on the actions taken in that department to justify this important decrease. However, there is a hypothesis that the actions conducted within the framework of the binational project were extended to the Nord Department.

For decades, the Sud-Est department has been the area with the lowest malaria burden in Haiti. From 2009 to 2018, the largest number of cases reported in this department was 989 in 2010, the year in which malaria had a significant rise in the country. A study conducted by Raccurt and collaborators^48^ demonstrated that malaria occurs in the form of heterogeneous foci in the coastal areas of the Sud department, with strong variations in the carrying rates of the gametocyte and/or trophozoite forms of *P. falciparum* from one locality to the other. 

The Sud-Est department, covering an area of ​​2,153 km², is composed of more than 65% of steep, mountainous regions^49^, including the highest mountain range in Haiti, the Massif de la Selle (2,600 m altitude). The plains, representing nearly 35% of the department’s total area, are all coastal, landlocked, and separated from each other by accentuated relief. The department is also characterized by its rainfall deficits, which have been recorded for decades (600 mm at most), and by frequent droughts^49^. The greatest proportion of the population in the department lives in the mountains. As the transmission of malaria in the southern region of Haiti occurs mainly in coastal areas^4^ ^,^ ^31^, the low incidence of malaria historically recorded in the Sud-Est department is likely because a significant portion of the departmental population at risk of contracting malaria is low.

This study revealed that in Haiti, official malaria data from 2009 to 2018 were reported by departments, rather than by municipalities. Given these facts, malaria risk should be assessed at the municipal level to target appropriate local interventions. Our results also indicate that some regions of Haiti are highly malaria-prone endemic areas. Therefore, additional studies are needed to assess the determinant factors associated with transmission dynamics and the high burden of the disease in these regions in Haiti. Such studies should analyze ecological determinants, such as landscape characteristics, demographic structures, and meteorological parameters.

This set of actions may help Haiti achieve the goal of eliminating malaria and preventing the reintroduction of the disease in areas where it can be eradicated.
